# Serum ANGPTL8 and ANGPTL3 as Predictors of Triglyceride Elevation in Adult Women

**DOI:** 10.3390/metabo12060539

**Published:** 2022-06-11

**Authors:** Anna Stefanska, Katarzyna Bergmann, Magdalena Krintus, Magdalena Kuligowska-Prusinska, Karolina Murawska, Grazyna Sypniewska

**Affiliations:** Department of Laboratory Medicine, Collegium Medicum, Bydgoszcz, Nicolaus Copernicus University, 87-110 Torun, Poland; bergmann@cm.umk.pl (K.B.); krintus@cm.umk.pl (M.K.); magda77@cm.umk.pl (M.K.-P.); murawska.karolina05@gmail.com (K.M.); odes@cm.umk.pl (G.S.)

**Keywords:** angiopoietin-like proteins, triglycerides, atherosclerotic cardiovascular disease, menopause

## Abstract

Angiopoietin-like proteins ANGPTL3 and ANGPTL8 have been shown to inhibit lipoprotein lipase, and thus regulate triglyceride level in the circulation. Whether the regulation of lipid metabolism by ANGPTLs is affected by the menopausal status remains unclear. We aimed to assess the relationships between serum ANGPTL3 and ANGPTL8 and atherogenic biomarkers in presumably healthy women during ageing. The study group included 94 women of whom 31 were premenopausal (PRE ≤ 40 years) and 37 were postmenopausal (POST ≥ 52 years). Atherogenic lipid and non-lipid biomarkers and ANGPTLs (ANGPTL3, ANGPTL8) were assayed in serum samples. TG/HDL-C index, non-HDL-cholesterol, remnant cholesterol concentrations, and BMI were calculated. Median levels of ANGPTL3 and concentrations of lipid biomarkers were significantly higher in POST comparing to PRE but ANGPTL8 levels were not different. In PRE, ANGPTL8 levels correlated significantly with TG and TG/HDL-C index while there were no correlations between ANGPTL3 and these biomarkers. In POST both ANGPTLs correlated with TG, sdLDL-C, and TG/HDL-C. ANGPTL8 and sd-LDL-C were the most significant predictors of early triglyceride elevation > 100 mg/dL (1.13 mmol/L) in the whole group and POST whereas the prediction power of ANGPTL3 was negligible in the whole group and non-significant in the subgroups. We demonstrated a significant positive correlation of ANGPTL3 with age category which predisposes to postmenopause. Despite the increase in ANGPTL3 level with ageing the ANGPTL3/ANGPL8 ratio was maintained. In conclusion, ANGPTL8 predicts the early triglyceride elevation better than ANGPTL3, especially in postmenopausal women. The association of ANGPTL3 with triglyceride levels is weaker than ANGPTL8 and depends on menopausal status. We suggest that the choice for the best efficient treatment of dyslipidemia with new inhibitors of angiopoietin-like proteins may depend on the menopausal status.

## 1. Introduction

Ageing with transition to menopause is one of the most important factors causing increased cardiovascular risk in women. Loss of ovarian hormones leads to dysregulation of lipid metabolism, mainly elevation of triglycerides (TG) in the circulation which appears a strong risk factor of atherosclerotic cardiovascular disease (ASCVD) [1]. The mechanisms associated with increased ASCVD risk in postmenopausal women are still unclear. Estrogens control lipid metabolism acting in the liver to decrease TG level and affect cholesterol metabolism. Estrogen signaling occurs through many pathways which are not well defined but loss of estrogen signaling adversely affects lipid metabolism [1]. Elevated TG levels, even in apparently healthy individuals, have been associated with the risk of cardiovascular diseases. Efficient metabolism of very low-density lipoproteins (VLDL) at optimal TG levels prevents accumulation of smaller triglyceride-rich remnants (TRLs) instead, elevation of TG and attenuated lipolysis in adipose tissue lead to increased generation of TRLs. Remnant lipoproteins may have a direct effect on atherosclerosis as they enter the arterial wall and cause the development of arterial plaque [2]. All TRLs contain cholesterol which is accumulated in the arterial wall and has proinflammatory effects [3,4]. Lipolysis of TG within TRLs by lipoprotein lipase (LPL), released from adipocytes, and translocated to the vascular endothelium, is a complicated process involving several activators and inhibitors [5].

Three angiopoietin-like proteins (ANGPTLs), ANGPTL3, ANGPTL4, and ANGPTL8, have been shown to inhibit LPL, and thus regulate TG levels in the circulation, depending on nutritional status [6]. In the fasting state, ANGPTL8 level is decreased but otherwise in the fed state its increased level activates ANGPTL3 which inhibits LPL [6,7]. Both ANGPTL3 and ANGPTL8 are expressed in the liver and could be secreted as a complex which enters into the circulation to inhibit LPL [8]. Previous studies have shown that inhibitory potential of ANGPTL3 towards LPL is enhanced when it is in complex with ANGPTL8, meaning that both ANGPTLs exert a coordinate action to regulate triglyceride metabolism [6]. ANGPTL8 is also expressed in the adipose tissue and recently was demonstrated in the visceral adipose tissue of adults [8,9]. Considering the fact that visceral adipose tissue lacks ANGPTL3 expression the physiological role of ANGPTL8 derived from visceral fat remains to be clarified. Currently, pharmacological and genetic lipid-lowering therapies are being tested targeting ANGPTL3 and ANGPTL8; therefore, investigations on ANGPTLs-mediated lipoprotein lipase inhibition continue to be of importance [10,11]. It is quite possible that therapies targeting ANGPTL8 and ANGPTL3 may have different outcomes in women and men.

Markedly decreased estrogen levels in postmenopausal women are regarded as the main factor for redistribution of body fat stores leading to increased amounts of visceral adipose tissue and diminished amounts in the femoral-gluteal area [12,13]. Whether the regulation of lipid metabolism by ANGPTLs is affected by the menopausal status remains unclear. We aimed to assess the relationships between serum ANGPTL3 and ANGPTL8 and atherogenic biomarkers in presumably healthy women during ageing with particular emphasis on evaluating the relationship between ANGPTLs and early TG elevation.

## 2. Results

The characteristics of study participants divided into age groups corresponding to menopausal status is displayed in Table 1. Overall, premenopausal (PRE) and postmenopausal (POST) women did not differ significantly in terms of body weight (BMI), high-density lipoprotein cholesterol (HDL-C), C-reactive protein (CRP), and ANGPTL8 concentrations. By contrast, median levels of ANGPTL3 and concentrations of lipid biomarkers were significantly higher in POST comparing to PRE women. Noteworthy, out of all lipid biomarkers, median TG and TG/HDL-C index were over two-fold higher in POST. Significantly higher concentrations of lipid biomarkers such as small-dense low-density lipoprotein cholesterol (sdLDL-C), TG, remnant cholesterol (remnant-C), and both ANGPTLs were found in perimenopausal (PERI) women compared to PRE. On the other hand, only total cholesterol (TC) and low-density lipoprotein cholesterol (LDL-C) levels were significantly higher when PERI and POST women were compared.

Correlation analysis between ANGPTLs and atherogenic lipid biomarkers performed in the whole study group indicated a highly significant positive association of ANGPTL3 and ANGPTL8 with TG (R = 0.375 and R = 0.528; *p* < 0.001), TG/HDL-C (R = 0.33; *p* = 0.001 and R = 0.52; *p* < 0.001), sdLDL-C (R = 0.29; *p* = 0.004 and R = 0.38; *p* < 0.001), and remnant-C levels (R = 0.25; *p* = 0.015 and R = 0.36; *p* < 0.001). In addition, ANGPTL3 was significantly related with TC (R = 0.305; *p* = 0.003) and apolipoprotein B (apoB) concentrations (R = 0.301; *p* = 0.003), whereas ANGPTL8 correlated weakly positively with CRP levels (R = 0.295; *p* = 0.004). 

Regarding menopausal status of women, the substantial differences pertaining to relationships between ANGPTLs and atherogenic lipid biomarkers were observed (Table 2). In PRE, ANGPTL8 levels correlated significantly with TG and TG/HDL-C index while there were no correlations between ANGPTL3 and these biomarkers. By contrast, in POST both ANGPTLs correlated with TG, sdLDL-C, and TG/HDL-C. Interestingly, in postmenopausal women a significant positive relationship appeared between ANGPTL3 and TC and apoB level.

In the course of further analysis, we developed logistic regression models for the prediction of TG elevation over the optimal value (>1.13 mmol/L; >100 mg/dL). The models for the whole group of women were age and BMI adjusted (Table 3). The multivariable analysis revealed that ANGPTL8 and sd-LDL-C were the best positive predictors of early elevation of triglycerides. The risk of TG elevation over the optimal value was higher by 46% per one unit increase of ANGPTL8 and by almost 22% higher in the case of sdLDL-C increase. Based on the results of Nagelkerke R2 the adjusted model with ANGPTL8 explained 44% of the variation for the occurrence of TG over 100 mg/dL and the one with sdLDL-C explained 71% of variation. The prediction power of ANGPTL8 for the occurrence of TG > 1.13 mmol/L (>100 mg/dL) was quite comparable to that for the occurrence of moderate hypertriglyceridemia > 1.69 mmol/L (>150 mg/dL) [OR (95% CI) per unit increase = 1.495 (1.225–1.826), *p* < 0.001, R2 Nagelkerke = 0.41]. By contrast, the prediction power of ANGPTL3 was negligible, although statistically significant.

Noteworthy, the unadjusted logistic regression models for the prediction of TG elevation over the optimal value (>1.13 mmol/L; >100 mg/dL) in POST showed similar results for ANGPTL8 [OR (95% CI) = 1.641 (1.009–2.667), *p* = 0.014; R2 Nagelkerke = 0.37] followed by sdLDL-C [OR (95% CI) = 1.352 (1.030–1.774), *p* = 0.028); R2 Nagelkerke = 0.70]. The prediction power of ANGPTL3 was not significant [OR (95% CI) = 1.009 (0.998–1.021), *p* = 0.078; R2 Nagelkerke = 0.13]. Similarly, the regression models developed in PRE were not significant. 

In our study group, 22.3% of women presented with CRP > 3.0 mg/L (>28.57 nmol/L), the indicator of low-grade inflammation. We assessed the association of both ANGPTLs with CRP > 3.0 mg/L in the logistic regression model, adjusted for BMI and age. The model developed for the whole study group revealed that ANGPTL3 slightly though significantly reduced the probability of CRP level above 3.0 mg/L (28.57 nmol/L) [OR (95% CI) = 0.990 (0.982–0.998), *p* = 0.009]. Despite the findings addressed above, a positive relationship between ANGPTL8 and CRP in Spearman’s correlation analysis, ANGPTL8 was not a predictor of CRP above 3.0 mg/L in logistic regression models adjusted for age and BMI [OR (95% CI) = 1.055 (0.951–1.169), *p* = 0.129].

In order to examine whether age categories which correspond to menopausal stages (PRE, PERI, POST), lipid biomarkers, and CRP are associated with ANGPTLs levels, and to evaluate the relationship between both ANGPTLs, we conducted stepwise multiple linear regression analysis (Table 4 and Table 5). In the multiple regression models ANGPTL3 and ANGPTL8 (as dependent variables) were correlated with age categories, CRP, and lipid biomarkers as explanatory variables. The final regression models were significant for both ANGPTLs (*p* < 0.0001) and explained 28% of ANGPTL3 (Table 4) and 36% of ANGPTL8 (Table 5) variation.

This analysis showed that TG levels are the most strongly and independently related with ANGPTL3 and ANGPTL8. Moreover, it showed that ANGPTL3 is negatively and significantly correlated with CRP (Table 4). The most important finding is, however, that ANGPTL3 was positively corelated with age category, which predisposes to postmenopause, irrespectively of TG and CRP levels (Table 4). Additionally, we found a significant negative relationship between both ANGPTLs in the created regression models.

The inverse relationship between both ANGPTLs prompted us to calculate the ANGPTL3/ANGPTL8 ratio and make a comparison between the two study subgroups. We found that median values of this ratio in PRE and POST women were not significantly different [42 (27–71) vs. 49 (28–76); *p* = 0.651, respectively]. This finding indicated that despite the increase in ANGPTL3 level with ageing, the relative proportion of both ANGPTLs was maintained.

## 3. Discussion

Ageing in women is accompanied by hormonal changes leading to disturbances in lipid metabolism and increased risk of cardiovascular diseases. Markedly decreased levels of estrogens in postmenopausal women and relative increase in androgens cause redistribution of body fat leading to enhanced accumulation of visceral abdominal adipose tissue [14]. In fact, despite similar BMI, postmenopausal women exhibited larger waist circumference than premenopausal women [15]. Increased lipolysis in visceral adipose tissue and enhanced delivery of fatty acids directly to the liver interferes with hepatic metabolism contributing to increased TG and VLDL synthesis [16]. The lipolysis rate in adipose tissue and the activity of lipoprotein lipase which hydrolyses triglycerides with uptake of fatty acids into adipocytes for re-esterification and storage as TG may differ in postmenopausal and premenopausal women [14,17].

Whether the regulation of lipoprotein lipase by ANGPTL3 and ANGPTL8 is affected by the menopausal status remains unclear; hence, we aimed to shed some light on this matter. Our study included apparently healthy women in a wide age range of 25–70 years and we compared the relationships between serum levels of ANGPTLs and atherogenic lipid biomarkers in premenopausal and postmenopausal women. While median concentration of ANGPTL3 was found to be significantly higher in POST than in PERI women, that of ANGPTL8 did not differ between the groups. 

Following stratification by menopausal status, significant positive correlations were observed between ANGPTL8 and selected lipid biomarkers TG, TG/HDL-C in both, PRE and POST women. Menopause is associated with increased cardiovascular risk due to adverse changes in lipid profile such as elevation of TG, sdLDL-C, and decrease in HDL-C level, characteristic features of atherogenic dyslipidemia [18]. In our study, only in POST women, a significant positive relationship was found between TG, sdLDL-C, and both ANGPTLs, which is in line with the finding that the association of ANGPTL3 with TG levels depends on menopausal status. Recent data reported a significant positive relationship between fasting serum ANGPTL8 levels and TG and a negative correlation with serum lipoprotein lipase (R = −0.438; *p* = 0.015) in non-diabetic healthy Japanese adults [19]. No correlation was found between ANGPTL8 and TC and HDL-C. Although the subjects in the referred study had in general lower BMI, TC, and TG, the results are in accordance with our observations. According to the authors, the relationship between ANGPTL8 and LPL may not be a causal one; therefore, further studies are necessary [19].

The lack of correlation between ANGPTL3 and TG observed in premenopausal women seems quite difficult to explain in light of the previous findings which suggested a coordinated action of both ANGPTLs towards inhibition of lipoprotein lipase [6]. We noticed, however, that in PRE and POST women ANGPTL3/ANGPTL8 ratio was not significantly different which indicated that the relative proportion of both ANGPTLs was maintained, irrespective of menopausal status.

It was demonstrated that ANGPTL3 may regulate not only triglyceride but also cholesterol metabolism [9]. In the present study, ANGPTL3 appeared to correlate with the levels of TC and the number of LDL particles, reflected by apoB concentrations. This, however, was observed only in the postmenopausal group in which TC, LDL-C, and apoB levels were significantly increased. During menopause, LDL-C from the circulation cannot be utilized as a substrate for estrogen synthesis [14]; therefore, we surmise a possible link between ANGPTL3 and cholesterol metabolism at menopause. 

A recent consensus statement from the European Atherosclerosis Society (EAS) defined TG concentrations as optimal (TG < 1.13 mmol/L; <100 mg/dL), borderline (TG 1.13–1.69 mmol/L; 100–150 mg/dL), and moderately elevated (TG 1.70–5.64 mmol/L; 151–500 mg/dL) [5] and underlined a causal relationship between atherosclerotic cardiovascular disease risk and increased levels of TG, VLDL, and TRL remnants. According to the consensus statement, accumulation of VLDL and TRL remnants in the blood starts at fasting TG > 100 mg/dL, whereas at TG levels over 1.69 mmol/L (150 mg/dL) ASCVD risk becomes clinically relevant. Hence, we looked for the best predictors of early elevation of TG, over the optimal value, bearing in mind the menopausal status of the women under study.

We found that ANGPTL8 and sd-LDL-C were the best significant positive predictors of early triglyceride elevation in the whole study group, and what is important, also in the POST women. The model with ANGPTL8, adjusted for BMI and age, and the unadjusted model for POST explained 44% and 31% of variation for the occurrence of TG over 100 mg/dL, per unit increase of ANGPTL8. It must be noted that the prediction power of ANGPTL8, as an early indicator of TG increase, was comparable to that for moderate hypertriglyceridemia (OR per unit increase = 1.495; *p* < 0.001). Interestingly, the prediction power of ANGPTL3 was negligible; moreover, in the model for POST it was not significant.

Multiple linear regression stepwise analysis allowed us to demonstrate that TG levels are the most strongly and positively related with both ANGPTL8 and ANGPTL3. Furthermore, we showed that ANGPTL3 was significantly negatively related with CRP. The inverse relationship between ANGPTL3 and CRP is not easy to explain. In a large population study of adults, with a predominance of men, it was indicated that ANGPTL3 levels were weakly positively correlated with inflammation, estimated by CRP values. This analysis was adjusted for several variables but not age and gender [20]. In our previous study we observed a significant positive correlation between ANGPTL3 and CRP and BMI in males whereas in females no relationship between these variables was found [21]. Certainly, the mechanisms linking ANGPTL3 and low-grade inflammation remain unclear and require further studies; however, we suggest that gender-dependent differences may contribute.

The most important finding was that ANGPTL3 was significantly positively related with age category which predisposes to postmenopause and this relationship was independent of TG and CRP concentrations. Since we found that the levels of ANGPTL8 were very similar in premenopausal and postmenopausal women, we could not expect any relationship with age categories predisposing to menopause.

To clarify these findings, it should be taken into account that ANGPTL3 in the circulation derives exclusively from the liver [7], whereas ANGPTL8 is secreted from the liver and likely from visceral adipocytes [7,8]. Lipid metabolism in the liver is, most of all, regulated by estrogens and this may suggest that ANGPTL3 level or function is in some way age-dependent, whereas that of ANGPTL8 is not. We may only suppose that at menopause changes in hepatic lipid metabolism lead to the overexpression of ANGPTL3 in the liver and in consequence to reduced metabolism of TG.

Although ANGPTL8 expression was shown to be higher in the liver than in adipose tissue, it is unknown whether ANGPTL8 in the circulation derives mainly from the liver or if the adipose tissue is also a source of this protein [8]. The data so far are controversial; some but not all reports demonstrated higher levels of ANGPTL8 in obese patients than in non-obese. In the previous study from our group, we noticed significantly higher levels of ANGPTL8 in apparently healthy women with obesity whereas ANGPTL3 levels were comparable [21]. 

An inverse, although very weak, relationship between ANGPTL3 and ANGPTL8 has been reported earlier in a large sample study of adults [20]. In the present study, the negative relationship between ANGPTLs was found in the multiple linear regression analysis but the proportion of both was similar, irrespective of menopausal status. Our findings do not exclude that both proteins are potent inhibitors of LPL action on triglycerides and we may only speculate whether they act jointly or not necessarily. Recent data indicate that, despite low potency of ANGPTL3 towards LPL, its overexpression may effectively reduce LPL activity even in the absence of ANGPTL8 [22]. This suggests that ANGPTL3, unless in complex with ANGPTL8, in physiological concentrations has little effect on blood triglyceride levels [23,24]. The complex of ANGPTL3 and ANGPTL8 inhibits LPL more strongly than either protein alone [9]. Active lipoprotein lipase is secreted from adipocytes and muscle cells and transported by the binding protein GPIHBP1 (glycosylphosphatidylinositol-anchored HDL-binding protein 1) to the surface of endothelial cells which, according to earlier reports, interferes with the inhibition of LPL by ANGPTL3 [25,26]. Therefore, the hypothesis arises that hepatic ANGPTL3 cannot be the only effective inhibitor of LPL in vivo [9].

This study has limitations. The sample size was relatively small; therefore, the subgroups of PRE and POST women were very small. However, we included only apparently healthy non-diabetic women and both subgroups had very similar glycemic status, CRP and HDL-C levels. We had no access to anthropometric measures and did not assess thyroid function but, referring to the recent data from our group, we may assume that most of the subjects were euthyroid [21]. The frequency of obesity was slightly higher in the postmenopausal than in premenopausal group (19% and 13%, respectively), but their BMI was comparable. Instead, the frequency of increased TG/HDL-ratio ≥ 3.5 which has been shown to be associated with insulin resistance, cardiometabolic risk, and cardiovascular disease in Caucasian populations [27] was three-fold higher in POST than in PRE women (32.4% vs. 9.7%). We believe this does not affect our results at first, as in our study the borderline correlation of ANGPTL3 with TG/HDL-C ratio was observed only in POST women (*p* = 0.055), secondly as the cut-off point for this ratio which may vary depending on the glycemic status [27] was similar in PRE and POST cases. We did not assess insulin resistance but, referring to our previous study in which both, TG/HDL-C index and HOMA-IR (the homeostasis model assessment-estimated insulin resistance) were calculated, it is safe to assume that in most women HOMA-IR was within the reference range [28]. Moreover, recent review articles reported that insulin does not alter ANGPTL8 levels in the circulation [9] and that the association of ANGPTL8 with insulin resistance and HbA1c in non-diabetic subjects seems contentious [29]. Furthermore, we did not find any relationship between ANGPTL3 or ANGPTL8 and HbA1c in PRE and POST women and in the whole study group as well (R = 0.089; *p* = 0.389 and R = 0.096; *p* = 0.355, respectively). Another limitation is the lack of information about the women’s menstrual cycles. To establish menopausal status, we used the age categories for the Polish population [30] and follicle stimulating hormone (FSH) concentration. All our postmenopausal women had a FSH concentration above 40 mIU/mL [31].

Moreover, we were not able to establish the causality responsible for the relationship between circulating ANGPTLs and atherogenic biomarkers. We only showed the statistical relationships between these parameters and suggested some potential mechanisms based on the recent literature review.

## 4. Materials and Methods

### 4.1. Study Participants

In total, 239 questionnaire-identified presumably healthy Caucasian women, aged between 24 and 84 years, were enrolled in a single-center, cross-sectional study [32]. Subjects were free from any heart disease, had no arterial hypertension or diabetes, underwent no cardiac intervention, and did not undergo cardiac or lipid-lowering therapy. Participants with infections and previously diagnosed chronic inflammatory disease were also excluded.

Premenopausal stage was defined as age below or equal to 40 years and postmenopausal as age above or equal to 52 years [29,30]. From a cohort of 239 women, a subset was selected for whom complete data on biochemical parameters and BMI were available Finally, 94 females (aged 25–70 years) were included in the study group of which 31 were premenopausal (PRE ≤ 40 years of age), 37 were postmenopausal (POST ≥ 52 years), and 26 were perimenopausal (PERI 41–51 years). Fasting venous blood samples were collected between 7.30 a.m. and 10.00 a.m. and serum was obtained. Anthropometric measurements were performed on the same day as blood samples were taken, and BMI (body mass index) was calculated.

All participants gave written consent to be included in the study. The study protocol was approved by the Local Bioethics Committee in accordance with the Helsinki declaration (KB 348/21.04.2015, annex KB 490/25.06.2019). 

### 4.2. Laboratory Measurements

Atherogenic lipid and non-lipid biomarkers, total cholesterol (TC), HDL-cholesterol (HDL-C), LDL-cholesterol (LDL-C), triglycerides (TG), and C-reactive protein (CRP) were measured in fresh serum, while apolipoprotein B (apoB) and small dense low-density lipoprotein cholesterol (sdLDL-C) were assayed in previously deep-frozen serum samples using an Abbott ARCHITECT ci8200 instrument (Abbott, Wiesbaden, Germany) and Pentra 400 analyzer (Horiba ABX, Montpellier, France). Reagents for the sdLDL-C assay were provided by Randox Laboratories. Serum FSH level was measured on an AxSYM (Abbott Diagnostics). TG/HDL-C index, non-HDL-cholesterol (non-HDL-C), and remnant cholesterol (remnant-C) concentrations and ANGPTL3/ANGPTL8 ratio were calculated as previously described [19]. According to the very recent EAS consensus definition the following cut-points for triglyceride levels were accepted: optimal ≤ 100 mg/dL (≤1.13 mmol/L), borderline 101–150 mg/dL (1.14–1.69 mmol/L), moderately elevated > 150–500 mg/dL (1.70–5.64 mmol/L) [5].

ANGPTL3 and ANGPTL8 determinations were performed in previously deep-frozen serum samples using BioVendor ELISA tests (BioVendor, Brno, Czech Republic), as previously described [19].

All measurements were performed at the Department of Laboratory Medicine, Nicolaus Copernicus University, Collegium Medicum in Bydgoszcz, Poland.

### 4.3. Statistical Analysis

The data were presented as medians and 25th and 75th percentiles (non-Gaussian distribution). The Shapiro–Wilk test was applied to test the normality. The variables were compared using the Mann–Whitney test. Spearman rank correlations were evaluated. Parameters with non-Gaussian distribution were normalized by natural log transformation for multiple linear regression and logistic analysis. In multiple linear regression analysis, age categories, CRP, and lipid biomarkers were assayed as independent variables and ANGPTLs as a dependent variable. In all the logistic models, odds ratios (ORs) were calculated for a 1 unit increase in independent variables. The significance of coefficients in the logistic models was tested using Wald chi-squared statistics. The goodness of fit of the models was evaluated using the Hosmer and Lemeshow chi-square test. To correct *p* values, the Benjamini–Hochberg procedure was applied to risk factor results to reduce the potential for type 1 errors. The level of statistical significance was set as 0.05 (Statistica 13.3, StatSoft Polska, Kraków, Poland or MedCalc statistical software, Ostend, Belgium).

## 5. Conclusions

In conclusion, we demonstrated that serum ANGPTL8 and sdLDL-C are good predictors of early triglyceride elevation, and better than ANGPTL3, particularly in postmenopausal women. The association of ANGPTL3 with triglyceride levels is weaker than ANGPTL8 and depends on menopausal status. These results indicate that the choice for the best efficient treatment of dyslipidemia with new inhibitors of angiopoietin-like proteins may depend on the menopausal status. As there are currently several new therapeutical approaches for the treatment of hypertriglyceridemia being assessed, we advocate that menopausal status should be taken into consideration when applying new therapies.

## Figures and Tables

**Table 1:** Characteristics of study participants dependent on menopausal status.

Variable	PRE ≤40 Years *n* = 31 (I)	PERI 41–51 Years *n* = 26 (II)	POST ≥52 Years *n* = 37 (III)	P I vs. II	P II vs. III	P I vs. III
Age [years]	36 (30–38)	46 (43–48)	56 (54–61)	<0.001	<0.001	<0.001
BMI [kg/m^2^]	24.4 (20.4–27.9)	27.7 (23.8–31.7)	25.2 (23.9–27.7)	0.014	0.194	0.085
TC [mmol/L]	4.91 (4.45–5.51)	5.48 (4.60–6.15)	6.21 (5.53–7.03)	0.099	0.014	<0.001
LDL-C [mmol/L]	2.95 (2.27–3.75)	3.14 (2.40–3.80)	3.77 (3.28–4.65)	0.548	0.011	<0.001
apoB [µmol/L]	1.71 (1.40–2.10)	1.83 (1.50–2.32)	2.16 (1.93–2.57)	0.214	0.055	<0.001
sdLDL-C [mmol/L]	0.64 (0.36–0.87)	0.97 (0.56–1.51)	0.98 (0.69–1.41)	0.011	0.839	0.001
TG [mmol/L]	0.82 (0.68–1.75)	1.99 (1.35–2.28)	1.70 (1.06–2.29)	0.001	0.485	0.001
HDL-C [mmol/L]	1.55 (1.29–1.68)	1.39 (1.19–1.58)	1.47 (1.34–1.68)	0.114	0.152	0.848
TG/HDL-C	1.33 (0.98–2.67)	3.26 (2.24–4.60)	2.67 (1.77–3.79)	0.002	0.201	0.005
Non-HDL-C [mmol/L]	3.49 (2.69–4.06)	4.28 (3.02–5.02)	4.55 (3.98–5.59)	0.058	0.051	<0.001
Remnant-C [mmol/L]	0.44 (0.31–0.54)	0.77 (0.54–1.01)	0.70 (0.52–0.88)	<0.001	0.539	<0.001
ANGPTL3 [ng/mL]	219 (178–245)	255 (211–301)	280 (208–335)	0.017	0.339	0.002
ANGPTL8 [ng/mL]	5.15 (3.10–7.71)	7.23 (4.61–9.49)	4.80 (3.97–9.48)	0.042	0.217	0.395
CRP [nmol/L]	7.33 (2.09–27.62)	20.38 (6.95–39.52)	7.81 (2.09–19.0)	0.047	0.020	0.801
FSH [mIU/mL]	5.0 (4.5–7.4)	12.7 (6.9–40.4)	57.9 (42.7–74.8)	0.001	0.001	<0.001

Medians (25th and 75th percentiles); BMI—body mass index; TC—total cholesterol; LDL-C—low density lipoprotein cholesterol, apoB—apolipoprotein B; sdLDL-C—small dense low density lipoprotein cholesterol; TG—triglycerides; HDL-C—high density lipoprotein cholesterol; Non-HDL-C—non high density lipoprotein cholesterol; Remnant-C—remnant cholesterol; ANGPTL3—angiopoietin-like protein 3; ANGPTL8—angiopoietin-like protein 8; CRP—C-reactive protein; FSH—follicle stimulating hormone; PRE—premenopausal women; PERI—perimenopausal women, POST—postmenopausal women.

**Table 2:** Spearman rank correlation coefficients between ANGPTL8 and ANGPTL3, and atherogenic lipid biomarkers.

**PRE *n* = 31**	**ANGPTL8**	**ANGPTL3**
TG	R = 0.437; *p* = 0.014	R = −0.099; *p* = 0.593
TG/HDL-C	R = 0.357; *p* = 0.048	R = −0.173; *p* = 0.351
**POST *n* = 37**	**ANGPTL8**	**ANGPTL3**
TG	R = 0.451; *p* = 0.005	R = 0.428; *p* = 0.008
TG/HDL-C	R = 0.494; *p* = 0.002	R = 0.317; *p* = 0.055
sdLDL-C	R = 0.391; *p* = 0.017	R = 0.394; *p* = 0.016
TC	R = 0.159; *p* = 0.345	R = 0.398; *p* = 0.015
apoB	R = 0.243; *p* = 0.146	R = 0.386; *p* = 0.018

PRE—premenopausal women; POST—postmenopausal women, the significant correlations are highlighted in gray.

**Table 3:** Multivariable logistic regression analysis for predictors of TG > 100 mg/dL (>1.13 mmol/L) in the study group.

Variable	Adjusted OR	95% CI	*p*	*p* *	Non-Adjusted NR^2^	Adjusted NR^2^
TC	1.032	1.018–1.048	<0.001	0.001	0.30	0.42
LDL-C	1.032	1.015–1.050	<0.001	0.001	0.26	0.40
apoB	1.083	1.046–1.121	<0.001	0.001	0.46	0.57
sdLDL-C	1.215	1.104–1.337	<0.001	0.001	0.68	0.71
Remnant-C	1.199	1.107–1.299	<0.001	0.001	0.52	0.55
ANGPTL3	1.013	1.005–1.021	0.02	0.02	0.13	0.27
ANGPTL8	1.464	1.191–1.801	<0.001	0.001	0.31	0.44

All models adjusted for age and BMI; OR—odds ratio, CI—confidence interval; NR2—Nagelkerke’s pseudo R2; *p* *—corrected *p*-value after applying Benjamini–Hochberg correction.

**Table 4:** The stepwise multiple linear regression analysis between ANGPTL3 and selected variables.

R2 = 0.28; *p* < 0.0001 *n* = 94				
Variables	β	S.E.	*p*	*p* *
Age categories	0.263	0.097	0.008	0.013
CRP	−0.278	0.095	0.004	0.01
TG	0.541	0.154	<0.001	0.005
ANGPTL8	−0.225	0.110	0.040	0.05
Remnant-C	−0.206	0.139	0.143	0.143

β—regression coefficient; S.E.—standard error; R2—multiple regression model R-squared; *p* *—corrected *p*-value after applying Benjamini–Hochberg correction.

**Table 5:** The stepwise multiple linear regression analysis between ANGPTL8 and selected variables.

R2 = 0.36; *p* < 0.0001 *n* = 94				
Variables	β	S.E.	*p*	*p* *
TG	0.704	0.108	<0.001	0.003
ANGPTL3	−0.219	0.088	0.015	0.022
sdLDL-C	−0.165	0.104	0.117	0.117

β—regression coefficient; S.E.—standard error, R2—multiple regression model R-squared; *p* *—corrected *p*-value after applying Benjamini–Hochberg correction.

## Data Availability

The data presented in this study are available on request from the corresponding author. The data are not publicly available due to privacy.
